# Normal aging and Parkinson's disease are associated with the functional decline of distinct frontal-striatal circuits

**DOI:** 10.1016/j.cortex.2017.05.020

**Published:** 2017-08

**Authors:** Aleksandra Gruszka, Adam Hampshire, Roger A. Barker, Adrian M. Owen

**Affiliations:** a Institute of Psychology, Jagiellonian University, Krakow, Poland; b The Division of Brain Sciences, Imperial College London, UK; c Cambridge Centre for Brain Repair, University of Cambridge, UK; d Department of Neurology, Addenbrooke's Hospital, Cambridge, UK; e The Brain and Mind Institute, University of Western Ontario, Canada

**Keywords:** Parkinson's disease, Aging, Functional fMRI, Frontostriatal circuitry, Attentional set-shifting, Learning

## Abstract

Impaired ability to shift attention between stimuli (i.e. shifting attentional ‘set’) is a well-established part of the dysexecutive syndrome in Parkinson's Disease (PD), nevertheless cognitive and neural bases of this deficit remain unclear. In this study, an fMRI-optimised variant of a classic paradigm for assessing attentional control (Hampshire and Owen 2006) was used to contrast activity in dissociable executive circuits in early-stage PD patients and controls. The results demonstrated that the neural basis of the executive performance impairments in PD is accompanied by hypoactivation within the striatum, anterior cingulate cortex (vACC), and inferior frontal sulcus (IFS) regions. By contrast, in aging it is associated with hypoactivation of the anterior insula/inferior frontal operculum (AI/FO) and the pre-supplementary motor area (preSMA). Between group behavioural differences were also observed; whereas normally aging individuals exhibited routine-problem solving deficits, PD patients demonstrated more global task learning deficits. These findings concur with recent research demonstrating model-based reinforcement learning deficits in PD and provide evidence that the AI/FO and IFS circuits are differentially impacted by PD and normal aging.

## 1 Introduction

Idiopathic Parkinson's Disease (PD) is a common neurodegenerative condition, in which the prevalent motor features, namely: bradykinesia, rigidity and resting tremor are frequently accompanied by impairments of executive functioning that closely resemble difficulties seen in clinical groups with known damage to frontal cortex. This so-called ‘dysexecutive syndrome’ is evident even from the early stages of the disease (Foltynie, Brayne, Robbins, & Barker, 2004) and includes deficits of working memory, planning, attentional control and set-shifting performance (Gotham et al., 1988, Grossman et al., 1992, Lees and Smith, 1983, Morris et al., 1988, Owen et al., 1992, Taylor et al., 1986). The exact neurochemical and neuroanatomical basis of these changes have yet to be clarified in PD. Executive dysfunction has previously been shown to be extremely sensitive to the effects of controlled levodopa (l-dopa) withdrawal (Lange et al., 1992), suggesting a predominantly dopaminergic substrate for the deficits observed. However, the relationship between dopamine and executive function is complex and the effect of dopamine replacement therapy on cognition often appears to be paradoxical (see: Cools, 2006 for review). Neuroimaging studies suggest that executive deficits in PD are accompanied by neural changes that are related to, but distinct from, those changes that underlie motor features (Lewis et al., 2003). The primary neuropathology of PD is dopaminergic neuronal loss in the nigrostriatal tract and also in the mesocortical pathway (Jellinger, 1991, Jellinger, 1999), which results in dopamine depletion within the frontal cortex itself (Scatton, Javoy-Agid, Rouquier, Dubois, & Agid, 1983). However, the mesocortical system is known to be less severely affected (50% depletion) than the nigrostriatal dopamine system (80% depletion) (Agid, Javoy-Agid, & Ruberg, 1987), and possibly, at a later stage of the disease process. Previous functional imaging studies exploring dysexecutive syndrome in PD have provided supporting evidence for a role of disruption in the nigrostriatal (Owen, Doyon, Dagher, Sadikot, & Evans, 1998), mesocortical (Cools et al., 2002, Mattay et al., 2002), or both of these pathways (Monchi, Petrides, Mejia-Constain, & Strafella, 2007), possibly depending on the extent to which the striatum is involved along with COMT genotype and drug therapy (Fallon et al., 2015, Williams-Gray et al., 2008).

Impaired ability to shift attention between stimuli (i.e. shifting attentional ‘set’) is a well-established part of the dysexecutive syndrome in PD with the deficits evident in both cognitive and motor domains (Cools et al., 1984, Downes et al., 1989, Owen et al., 1992, Van Spaendonck et al., 1996). However, the psychological, neurochemical and neuroanatomical bases of this deficit remain unclear. In the cognitive domain, attentional set-shifting performance in PD has been studied most extensively using tests of visual discrimination learning, such as the Wisconsin Card Sorting Test (Berg, 1948) and the CANTAB ID/ED task (Downes et al., 1989, Owen et al., 1992). Using this paradigm, a number of studies have shown that PD patients, like patients with frontal-lobe damage, are more impaired when a so-called ‘extradimensional shift’ (EDS) is required (i.e., a switch between two competing perceptual dimensions such as ‘colour’ and ‘number’), than when a so-called ‘intra-dimensional shift’ (IDS) is required (i.e., a switch between two different values of the same dimension such as ‘blue’ and ‘red’) (Roberts, Robbins, & Everitt, 1988). However, tasks based on visual discrimination traditionally used to assess set-shifting performance, have been criticized for their low cognitive resolution, i.e., for confounding multiple cognitive processes (e.g., the greater demands placed on working memory and novel rule learning during the EDS versus the IDS) (Hampshire & Owen, 2006; Ravizza & Ciranni, 2002). Moreover, in pharmacological studies, mixed results have been reported with regard to the role of dopamine levels in set shifting in PD (Cools, Barker, Sahakian, & Robbins, 2001; Hayes et al., 1998, Lewis et al., 2005, Owen et al., 1993, Ravizza and Ciranni, 2002, Slabosz et al., 2006) suggesting that nondopaminergic forms of pathology may also contribute to these impairments (Kehagia et al., 2010, Lewis et al., 2005).

The neuroanatomical basis of attentional shifting deficits in PD is also uncertain. Neuroimaging studies in healthy controls have associated set-shifting performance with increased activation of the prefrontal cortex (PFC) (Cools et al., 2002, Dove et al., 2000, Konishi et al., 1998, Ravizza and Ciranni, 2002, Rogers et al., 2000) and interconnected posterior cortical systems (Hampshire & Owen, 2006). Several neuroimaging studies have implicated the caudate nuclei in set-shifting (Monchi et al., 2001, Monchi et al., 2006, Stewart et al., 2001). Similar patterns of set-shifting impairments to those observed in PD (Downes et al., 1989, Owen et al., 1993) have also been observed in patients with known damage to the PFC (Owen et al., 1993, Rogers et al., 1998) and patients that have known basal ganglia pathology, i.e., Huntington's disease (Lawrence et al., 1996). Moreover, damage to different regions of the caudate nucleus in non-human species produces deficits that often resemble the effects of damage to their corresponding targets of projection within the PFC (Divac, Rosvold, & Szwarcbart, 1967), and 18F-dopa PET studies in PD patients have shown a correlation between dopaminergic depletion of the caudate nucleus and neuropsychological performance (Marié et al., 1999). Thus, the available evidence broadly suggests that attentional set-shifting performance is mediated by the combined operation of frontocortical and subcortical mechanisms, possibly involving discrete frontostriatal ‘loops’ routed from various areas of the PFC, through the striatum, pallidal and thalamic nuclei back to the originating prefrontal region (Alexander, DeLong, & Strick, 1986). Consequently, it is unclear whether attentional set-shifting deficits in PD arise predominantly through their cortical (frontal lobe) or subcortical (striatal dopamine depletion) damage, which effectively interrupts the normal flow of information through frontostriatal circuitry (Owen et al., 1998). Moreover it is unclear, which frontal-striatal circuits are affected in PD.

The potentially conflicting results of cognitive neuroimaging studies in PD may reflect several methodological confounds. Most importantly, these studies have used tasks with relatively low psychological resolution (e.g. WCST) (Monchi et al., 2004). Moreover, rather than being triggered internally as a series of self-directed shifts in search of optimal responses, the behaviours under examination were driven by external cues indicating that shifts of attention were required and consequently, may not be the most valid test of ‘executive’ function.

**Fig. 1:**
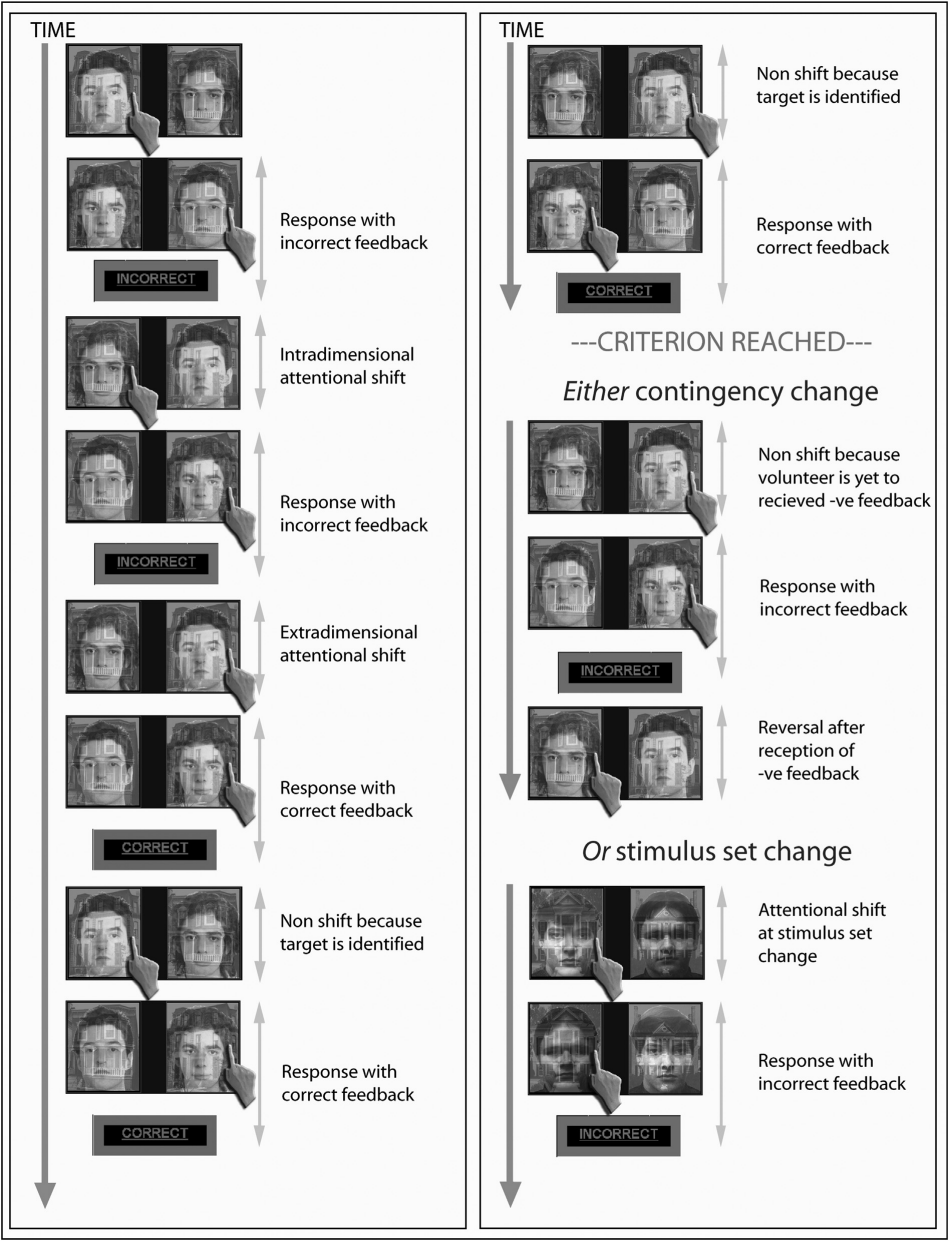
**Experimental design**. On each trial, volunteers looked at two images presented on the screen, each of which comprised a face and a house superimposed. The volunteers' task was to work out through trial and error which object (face or house) was the target item. If volunteers supposed that the left-hand stimulus contained the correct object (as shown in this example), they pressed the left button, and vice versa. After every second response, relevant feedback was presented on the screen (“CORRECT” or “INCORRECT”). Once a criterion of six consecutive correct responses was reached, either the correct object was changed or a new stimulus set was presented; the volunteer was then required to learn the new correct object (Hampshire & Owen 2006; Hampshire et al., 2008).

The aim of the present study was to define the neural basis of attentional set-shifting deficits in PD by employing an fMRI-optimised variant (Hampshire & Owen, 2006) of the classic ID/ED attentional set-shifting paradigm (Roberts et al., 1988), with a proven sensitivity and specificity to the involvement of discrete neural substrates in separate cognitive components of attentional control (see: Hampshire & Owen, 2006 for the details). In this task, the subject is required to work out which of the two objects is the ‘target’ in a visual stimulus set by performing a serious of self-directed visual discriminations that involve ID (e.g., face to face) or ED (e.g., face to building) shifting. Each object consists of two compound stimuli (each composed of a face and a building superimposed on top of each other) (see Fig. 1).

The task allows distinct components of attentional control to be teased apart (i.e., responding to novel stimuli, IDS and EDS, overriding the response to a previously relevant stimulus, and responding to positive feedback), avoiding confounds inherent in the original CANTAB ID/ED paradigm. Furthermore, the task can be used to quantify the individual's chosen problem solving strategy by monitoring the focus of attention rather than imposing attentional switching externally. The task has been validated as a tool for fractionating these processes behaviourally and also at the level of brain activity in young (Hampshire & Owen, 2006) and elderly (Hampshire, Gruszka, Fallon, & Owen, 2008) healthy controls, as well as neuropsychological populations (Chamberlain et al., 2008; Williams-Gray, et al., 2008). In the current study, we used this protocol to explore the neural basis of attentional set-shifting deficits in PD on multiple discrete executive circuits. Based on previous work (Williams-Gray et al., 2008), it was hypothesized that abnormalities in PD will be observed primarily in the networks that underlie either reversal learning or extra-dimensional set shifting. More specifically, it was expected that in the patients group the task performance will be related to underactivity within the dorsal fronto-parietal networks, and given the nature of the PD pathology, within the fronto-striatal activity.

## 2 Materials and methods

### 2.1 Participants

#### 2.1.1 Patients

Eighteen right-handed patients (mean age *M* = 62.39, SD = 9.31, 7 females) with idiopathic PD were included in this study. All were in the early stages of the disease (Hoehn and Yahr grades I–II; Hoehn & Yahr, 1967). The group was drawn from a pool of the Parkinson's disease Research Clinic at the Cambridge Centre for Brain Repair where they had undergone careful historical review along with physical examination and neuropsychometric analysis. All patients satisfied UKPDS Brain Bank criteria (Gibb & Lees, 1988), were non-demented, with no current depressive illness, and no history of other neurological or psychiatric disease (Table 1). All testing was performed with patients taking their usual medications. Each participant's current dopaminergic drug regime was recorded and converted to an equivalent levodopa dose (Williams-Gray et al., 2008). None of the patients were taking acetylcholinesterase inhibitors.

**Table 1:** Demographic and clinical characteristics.

Variable	PD (*N* = 18)	CS (*N* = 16)
Age (y)	62.39 (±) 9.32	59.75 (±) 8.04
NART	114.33 (±) 10.85	117.40 (±) 8.05
BDI-II	9.37 (±) 6.32	10.00 (±) 5.63
UPDRS	29.50 (±) 17.20	
H&Y	1.86 (±) .69	
Years since diagnosis	4.77 (±) 1.37	
l-dopa (daily, mg)	347.22 (±) 397.96	

PD – patients with PD, CS – age matched control group, NART – the National Adult Reading Test (Nelson, 1982), BDI-II – Beck Depression Inventory II (Beck et al., 1996), UPDRS – Unified Parkinson's Disease Rating Scale (1987), H&Y – Hoehn and Yahr scale (Hoehn & Yahr, 1967). Values represent mean ± SD of the mean. Between-group comparisons using Student's *t* test revealed no significant differences (*p* > .05).

#### 2.1.2 Healthy volunteers

A group of healthy controls matched as closely as possible to the PD group with respect to age and pre-morbid verbal IQ as assessed by the National Adult Reading Test (NART; Nelson, 1982) was also recruited. The sixteen healthy subjects who participated were recruited from the volunteer panel at the MRC Cognition and Brain Sciences Unit (mean age *M* = 59.75, SD = 8.04, 10 females). They had no history of neurological or psychiatric disease. There were no significant differences between the patient and control group with respect to age, NART or Beck Depression Inventory II (BDI, Beck, Steer, Ball, & Ranieri, 1996) (Table 1). Ethical approval for the study was obtained from the local research ethics committee and all subjects gave their written informed consent.

### 2.2 Experimental design

A full description of the set-shifting task used in the present study has been published elsewhere (Hampshire and Owen, 2006). In the task volunteers had to work out which object was the ‘target’ in a stimulus set using task feedback (Fig. 1). The stimulus set consisted of two compound stimulus pairs appearing on the left and right of the screen. Both pairs were composed of a face and a building superimposed on top of each other. Each stimulus subtended a visual vertical angle of 6° and a horizontal angle of 6.2°, with a total combined horizontal angle of 15°. On each trial, the volunteers were required to indicate using a button box which side of the screen they thought the target was located on. This response triggered the removal of the stimuli from the screen. Every second response, feedback was presented on the screen for .6 sec, indicating whether the stimulus they had chosen was the target or not. The feedback given was the word “CORRECT” in green if the last two responses were both correct. Otherwise, the feedback was the word “INCORRECT” in red.

After six correct responses to the target (that is, three positive feedback events) a change of target occurred. The change was either in the form of a set change, in which new compound stimulus pairs were presented, or a reward contingency change, in which the set would stay the same and a previous non-target would become the target (due to a rule change). In either case, at this moment the subjects selected a new target performing an ID shift or an ED shift due to the set change (i.e., IDS EDS, respectively) or due to the reward contingency change (i.e., IDR or EDR, respectively). Thus, the task allowed to compare between different types of switch: made based on a change in the stimuli and made based on a change in the reward contingency. Maximum uncertainty was ensured in both cases, as the new target could be either a stimulus of the same category or a stimulus of the alternative category. As the face-house combinations comprising the compound stimuli were reversed on every trial, it was possible to calculate exactly which stimulus was being attended to by examining consecutive responses. The partial feedback technique also allowed the response events that comprised attentional switch decisions (first response) to be modelled separately from those confounding response with feedback (second response).

The experimental acquisition consisted of two 15-minute runs. As the task was response-driven, the number of switches completed varied for each volunteer. The inter-stimulus-interval was randomly jittered from .6 to 1.6 sec. Volunteers also underwent a pre-scanner training session for two ten-minute blocks to ensure they understood and were capable of performing the task prior to entering the scanner. Since the experimental task was set up in a way that 60 consecutive incorrect responses (i.e., 30 consecutive negative feedback events) resulted in the premature ending, completion of full two blocks ensured that the participants were familiar with the task and were able to perform the task at least at the most general level. Responses were made using the first and second fingers of the right hand on a button box. Response times (RTs) and the number of errors were recorded throughout the experimental acquisition. The volunteers were explicitly instructed to keep responding to the correct target until informed that it was no longer the target. They were also asked to respond ‘as quickly and accurately as possible’. Although it is possible that volunteers could compute the number of trials required to reach criteria and then make anticipatory switches during reversals, the performance data confirmed that this never actually happened.

### 2.3 Event modelling

The event modelling focused on individual types of volunteer response on a trial-by-trial basis, defined according to the current and previous foci of attention. There were five types of switch event, one non-switch event, and the responses with positive and negative feedback during solution search and when the target was known (Fig. 1).

Two of the switch events related to the period when the volunteer was actively trying to work out which was the target; one was termed ‘extra-dimensional’ because the focus of attention switched between stimuli of different types (for example, from a face to a building) and the other ‘intra-dimensional’, because the focus of attention switched between stimuli of the same type (for example, from one face to another face). Whilst each of these events involved multiple switch components (for example, response suppression and attended stimulus change), the only way in which they differed from one another was with respect to the change of attention to stimulus type, so subtraction of one from the other isolated this ED component. Two additional switch events were defined at the point when the volunteer had correctly identified the previous target and a different stimulus became the new target. In one of these switch events, the stimulus set was changed so the volunteer could not respond to the previous target, but had to switch to a target that had not been seen previously. This effectively removed any response suppression component and was called a ‘set change’. In the other switch event, the stimulus set stayed the same but the reward contingency changed. Thus, a negative feedback event to the previous target occurred, and the volunteer was required to shift attention to look for the new target. Because the new target was a previous non-target and because the previous target was still present (but as a non-target), this manipulation was termed a ‘reversal’. Whilst these two events had multiple components, subtraction of switching with stimulus set change from switching with reward contingency allowed examination of the reversal aspect of attentional shifting.

The final switch event was the first response to the correct target after the volunteer had received positive feedback. At this stage an important behavioural change occurred as the volunteer stopped trying to work out which was the target (solution search) and began to respond to the stimulus that they now knew to be correct. This switch corresponds to what the volunteer was doing rather than what they were attending to, which remained the same. This event was compared to the otherwise identical subsequent events (the sixth event type), in which the responses were made to the same stimulus again whilst knowing it was correct on the basis of feedback, and here these are called early and late correct responses. Contrasting these two events therefore isolated the goal change component of cognitive control; that is where the volunteer changes their behavioural focus from identifying which stimulus is the target to identifying the location of the known target.

Finally, positive and negative feedback events were compared directly to isolate any components involved specifically in processing the reception of abstract positive and negative rewards.

### 2.4 Imaging acquisition

The 18 early stage PD patients, and 16 age matched volunteers were scanned at the Wolfson Brain Imaging Centre using a 3 T Bruker Medspec scanner (Bruker s300, Ettingen, Germany) with 21 slices (4 mm slices with 1 mm inter-slice gap) per image and a TR of 1.1 sec and in plane resolution of 3.125 × 3.125 mm 850 T2-weighted echo-planar images, depicting BOLD contrast were acquired per run, and the first 18 were discarded to avoid T1 equilibrium effects. Images were slice time acquisition corrected, reoriented, subject motion corrected, geometrically undistorted using phase maps (Cusack, Brett, & Osswald, 2003), spatially normalised to the standard Montreal Neurological Institute EPI template, smoothed with an 8 mm full-width at half-maximum Gaussian kernel, and modelled using SPM (Wellcome Department of Cognitive Neurology).

### 2.5 Imaging analysis

Single subject statistical contrasts were set up by using the general linear model in SPM to fit each voxel with a combination of functions derived by convolving the standard haemodynamic response with the time series of the events, removing low-frequency noise with a high-pass filter. For switch events, durations were measured from stimulus onset to response at which stage the stimuli were removed from the screen, whereas feedback events were modelled by feedback display time. Images depicting the contrasts of interest were generated at the individual participant level and exported for group level analyses. Cross-group comparisons controlled for false positives using whole brain FWE cluster level correction set to *p* < .05 with robust permutation modelling in the Cambridge Brain Analysis software suite (Bullmore et al., 1999).

## 3 Results

### 3.1 Behavioural analysis

Three different behavioural measures were taken. First, the total number of targets identified was used as a rough overall measure of performance. Secondly, the number of errors made whilst searching for the target under different conditions of possible target change, namely: ID shifts following a set-change (IDS), ID shifts following reversal of reward contingency (IDR), ED shifts following a set-change (EDS) and ED shifts following reversal of reward contingency (EDR) were recorded. Thirdly, mean RTs were recorded for each of the types of subject response, namely: ID and ED shifts committed both while working out the correct target, first response following a set change, first response following a reversal of reward contingency, first correct response to a target, and late correct response to a target.

#### 3.1.1 Overall performance

**Fig. 2:**
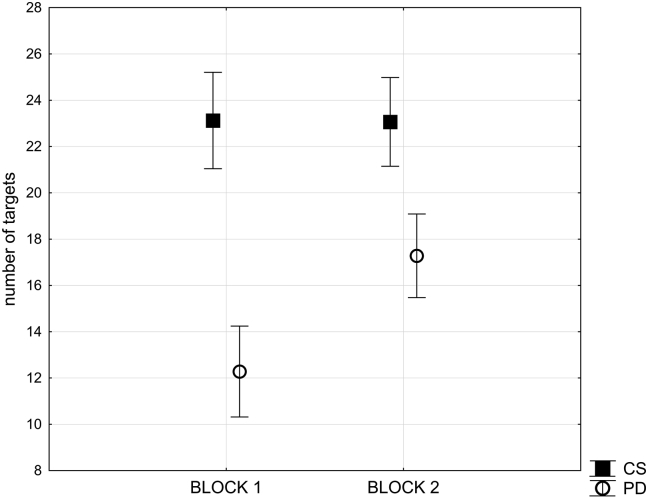
**Effect of PD pathology on overall performance**. This figure illustrates the mean number of targets identified for block one and block two of the experimental task compared across the patients with PD and the matched control group (CS). Bars represent standard error of the mean.

Controls had clearly reached a learning asymptote by the time they started the experiment within the scanner, correctly identifying an average of 23 targets in both block one and in block two. However, the PD group appeared to still be acquiring the task in the first experimental block, with an average of 12.03 targets correctly identified in block one and 17.03 in block two. This difference was examined in an ANOVA in which the within subject factor was block (one or two), and the between subject factor was group (PD or control) (Fig. 2). There was a significant interaction of group by block (*F*_(1, 32)_ = 9.1, *p* < .005). There was also a significant main effect of group (*F*_(1, 32)_ = 10.1, *p* < .003). An independent measures *t*-test revealed that there was still a significant effect of group within block two (*t*_(1, 32)_ = 3.2, *p* < .005), with the PD patients identifying fewer targets.

#### 3.1.2 Error rates

**Fig. 3:**
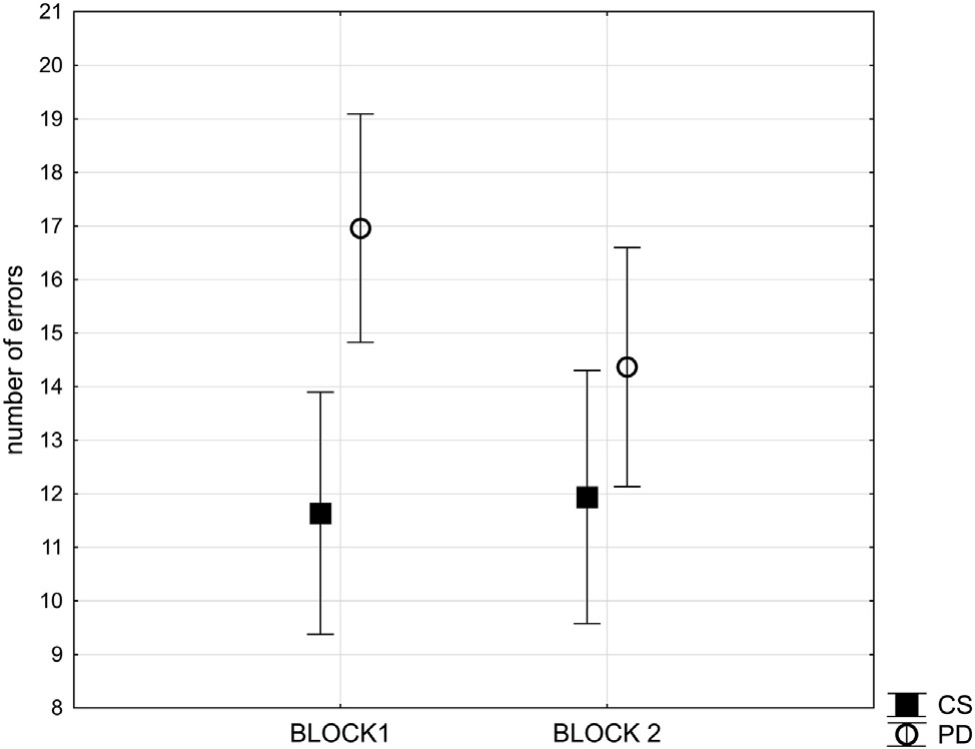
**Effect of PD pathology on error rate**. This figure illustrates mean number of error rates compared across the patients with PD and the matched control group (CS) for block one and block two the experimental task. Bars represent standard error of the mean.

As the PD group and the age-matched group appeared to differ in their performance with respect to the block of the task, this difference was further examined with respect to the four types of target change by analysing the number of errors committed before correct target identification using a 2*2*2*2 multi-way repeated-measures analysis of variance. The first factor was dimension change (ID or ED), and the second factor was reversal factor (whether the target changed with reward contingency change or stimulus set change). The third factor was experimental block (one or two). The fourth factor – group (PD or age matched control) – was included as a between subject variable. It was again clear that the PD group was impaired at acquiring the task compared with controls, with a significant interaction of experimental block by group (*F*_(1, 32)_ = 7.6, *p* = .01) (Fig. 3). The analysis revealed also significant main effects of group (*F*_(1, 32)_ = 6.7, *p* < .05), block (*F*_(1,32)_ = 4.7, *p* = .04), and reversal factor (*F*_(1,32)_ = 22.7; *p* < .001). No other significant effects due to either variable were observed. Table 2 presents mean number of errors per condition per block observed in the group of the patients with PD and in the control group (see also Supplementary Fig. 3a).

**Table 2:** Mean number of errors made for condition of a given type compared across the patients with PD (PD) and the matched control group (CS).

	Block 1				Block 2			
	IDS	EDS	IDR	EDR	IDS	EDS	IDR	EDR
CS	10.52 (1.35)	11.36 (1.36)	12.09 (1.54)	12.57 (1.43)	11.04 (1.08)	11.03 (1.24)	12.52 (1.59)	13.17 (1.39)
PD	16.36 (1.27)	14.92 (1.28)	18.29 (1.45)	18.26 (1.35)	12.54 (1.02)	14.15 (1.17)	15.53 (1.50)	15.251.31

PD – patients with PD, CS – age matched control group, IDS – intra-dimensional shift following a set-change, EDS – extra-dimensional shift following a set-change, IDR – intra-dimensional shift following a reversal of reward contingency, EDR – extra-dimensional shift following a reversal of reward contingency. Values represent: mean (standard error of the mean).

The behavioural data from the block two only was further compared across groups (PD or age matched control) with respect to the four types of target change (IDS, EDS, IDR, EDR). This analysis revealed no significant main effects or interactions of group. Within block two there was a significant main effect of reversal factor (*F*_(1, 32)_ = 15.4, *p* = .001) with more errors being committed under the reversal condition as compared to the set-change condition. No significant main effect of ED versus ID shifting was observed. Table 2 presents mean number of errors per condition for block two observed in the group of the patients with PD and in the control group.

Finally, a supplementary analysis was conducted to examine which particular aspect of the performance of the PD group had changed from the first to the second block. Thus, a three-way ANOVA of block (one or two), dimension change (ID or ID) and reversal factor (shifts due to set change or reversal of reward contingency) for the PD group only was performed. This analysis revealed significant main effects of block (*F*_(1, 17)_ = 10.8, *p* = .004) and the reversal factor (*F*_(1, 17)_ = 11.2, *p* = .004). However, it revealed no significant interactions among factors, suggesting that the change in performance in the PD group was non-specific. Relevant mean error rates per condition for the patients with PD regarding this interaction can be found in Table 2.

#### 3.1.3 RTs

**Fig. 4:**
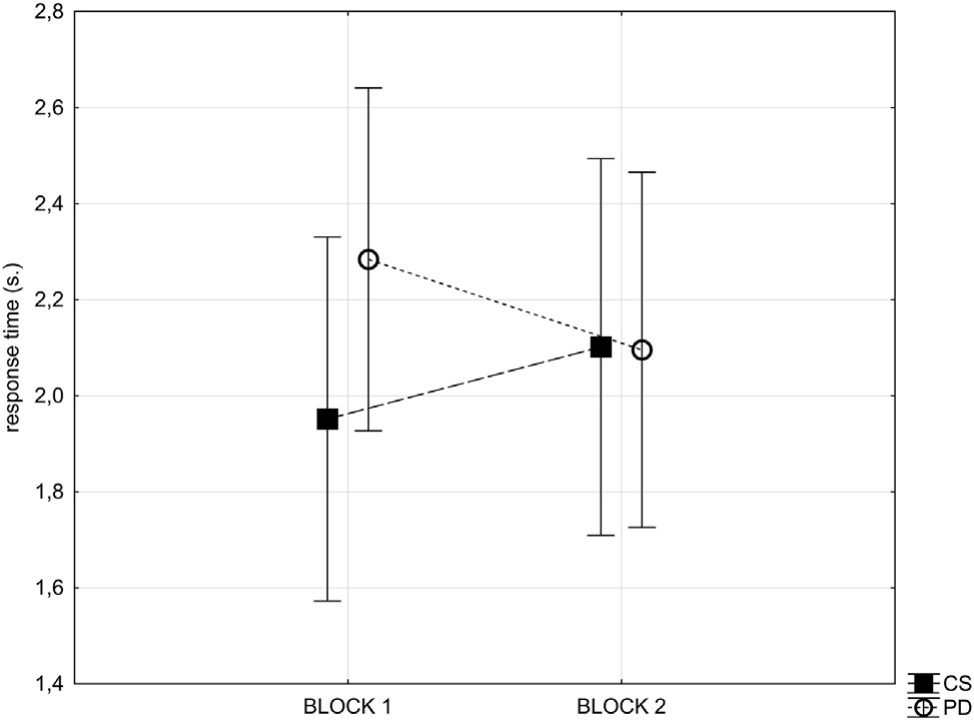
**Effects of PD on** RTs. This figure illustrates the RTs compared across the patients with PD and the matched control group (CS) for block one and block two of the experimental task. The group with PD displayed a trend towards slower response in block one. Bars represent standard error of the mean.

The RTs were compared for the individual response types that were subsequently modelled in the fMRI analysis to give an indication of their comparative difficulty in a 6*2 repeated measures ANOVA. The first factor was response type, and the conditions were: ED, ID, reward contingency change, stimulus set change, first known correct response, subsequent known correct response. The second factor was experimental block (first and second), and group (PD or age matched control) was included as a between subjects factor. In concordance with the error data, there was a significant interaction of experimental block by group (*F*_(1, 32)_ = 10.2, *p* = .003), with no significant main effect of group, and a trend towards slower response for the PD group in the first experimental block (Fig. 4).

**Fig. 5:**
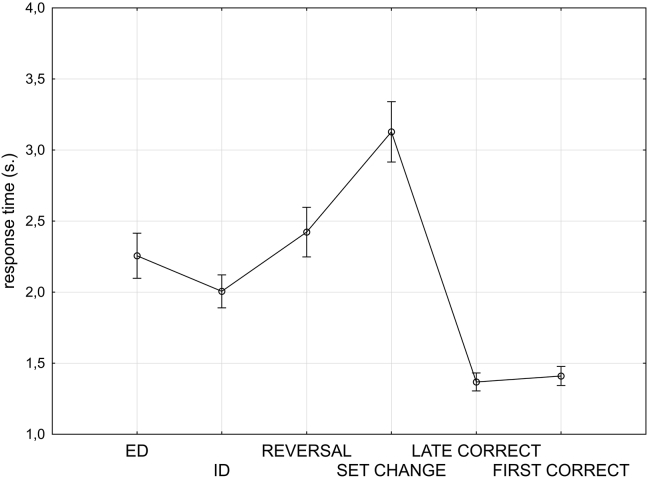
**Effects of the six types of response events on** RTs. Comparison of groups on reaction times for block two only of the experimental task revealed no significant effect of PD pathology. Bars represent standard error of the mean.

RTs data for just the second block of the task, when PD patients were also at the learning asymptote, revealed no significant effect of group, and a significant effect of response type (*F*_(5, 160)_ = 44.156, *p* < .001) (Fig. 5). Supplementary Fig. 5a depicts an insignificant interaction of group and response type.

As the group factor had no significant effect on reaction times during the second block performance, to examine the overall effect of response type, RTs data collapsed across groups in relation to the response type for block two only were analysed (Fig. 5). Pair-wise comparisons of the RTs for block two revealed that volunteers were slower when they decided to move their attention between, rather than within, stimulus dimensions (*t*_(1, 33)_ = 2.9, *p* = .005), and slower when moving attention within dimensions than when routinely responding to the known target (late correct responses) (*t*_(1, 33)_ = 7.6, *p* < .001). Furthermore, shifts of attention due to set change were compared with those due to reversal of reward contingency. In direct contrast to the error data described above (where more errors were made in the blocks following reward contingency change), the results revealed a significantly greater RTs for the set change condition (*t*_(1, 33)_ = 3.6, *p* < .001). There were no significant differences between the early and late correct responses (i.e. following the first positive feedback events versus those subsequent).

#### 3.1.4 Effects of medication on PD group performance

**Fig. 6:**
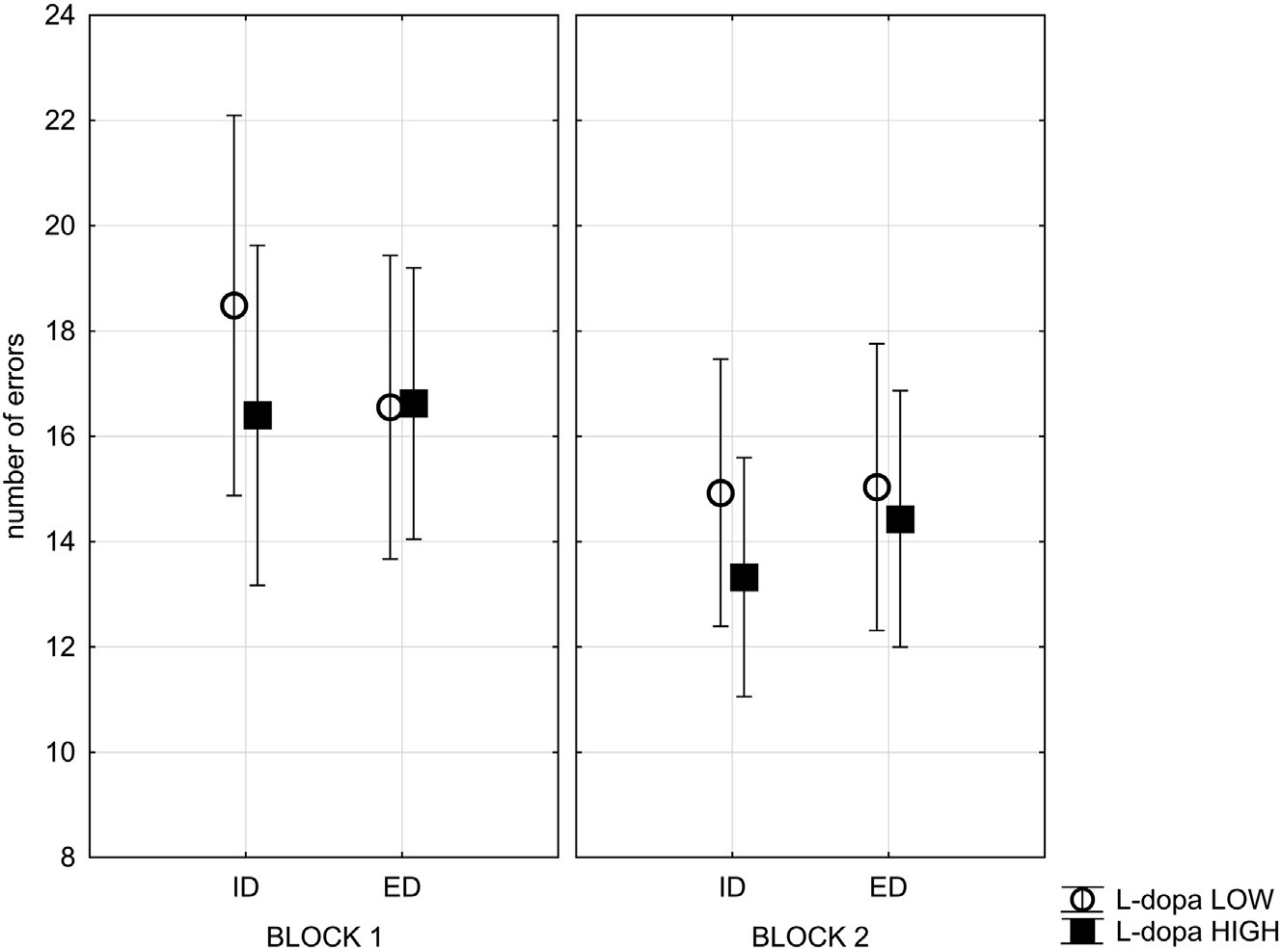
**Effects of****l****-dopa on error rate**. This figure illustrates the effects of l-dopa dose on the mean number of errors per type of target change (ID or ED) for both the first and the second block of the task. Significant predictor l-dopa dose was broken down by median-split for visualization purpose only. Bars represent standard error of the mean.

The effect of l-dopa on the performance of the PD group was investigated with 2*2*2*2 GLM Repeated Measures Model of block, dimension change and reversal factor. l-dopa dose was specified as a covariate. This revealed no significant main effect of l-dopa dose. However, a 2-way interaction of l-dopa dose and dimension change was significant (*F*_(1, 16)_ = 4.8, *p* = .05) as well as a 3-way interaction of l-dopa, block and dimension change (*F*_(1, 16)_ = 4.5, *p* = .05) (see Fig. 6). This interaction suggests that the patients on low levels of l-dopa committed higher number of IDS errors, particularly during the first block of the task. Finally, the effects of l-dopa on the RTs of the performance of the patients was tested. The 3-way ANOVA of l-dopa, block and response type revealed neither a main effect of l-dopa, nor any significant interactions (Supplementary Fig. 6a).

### 3.2 Results – Functional Imaging Analysis

Due to the block*group behavioural effects, the event related fMRI analysis focused upon only the second task block, when the two subject groups had learnt the task and were performing at a similar level of competence. Four contrasts were examined in all subjects, collapsing across the patients and controls in order to replicate previous findings (Hampshire & Owen, 2006) regarding which brain regions were recruited during which stages of task. Subsequently, cross group analyses were conducted for the same contrasts, in order to identify which brain regions were affected under which conditions in PD relative to controls.

#### 3.2.1 Solution search versus routine responding

**Fig. 7:**
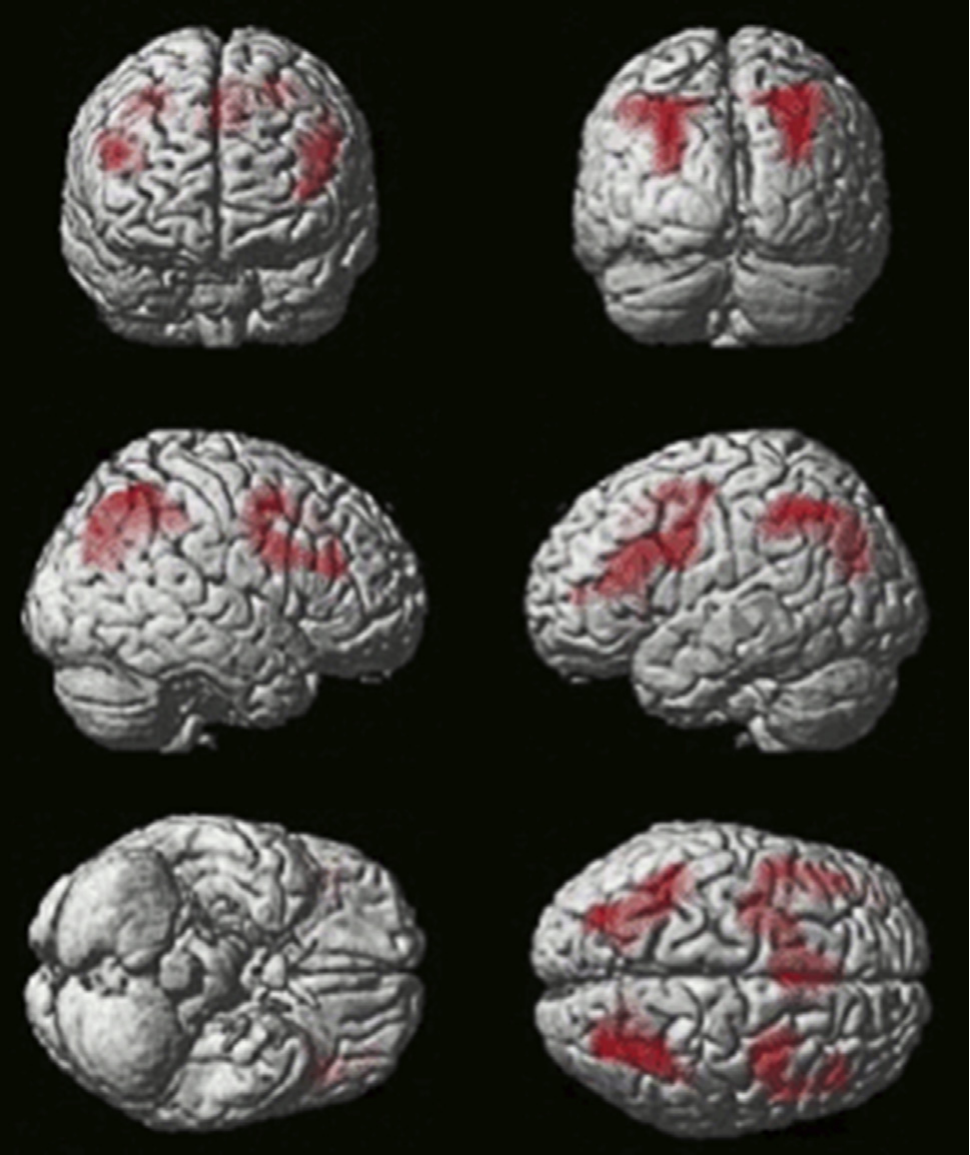
**Activation during ‘working out’ versus ‘known correct’ events collapsed across all subjects (*N*** = **34)**. (Thresholded voxel-wise at *p* < .01 with false positive controlled for across the whole brain mass at *p* < .05 using FWE cluster correction in SPM5).

To localise the neural correlates of solution search, all events where the target was known (early and late correct responses, and feedback events whilst the target was known) were subtracted from all events where the volunteer was actively trying to work out the target (extra-dimensional and intra-dimensional shifts, reversals, set change, and feedback events during solution search). In line with the previous study by Hampshire and Owen (2006), collapsing across groups revealed significant solution search related activity bilaterally in the inferior frontal sulcus (IFS) and the posterior parietal cortex (PPC) (Fig. 7).

**Fig. 8:**
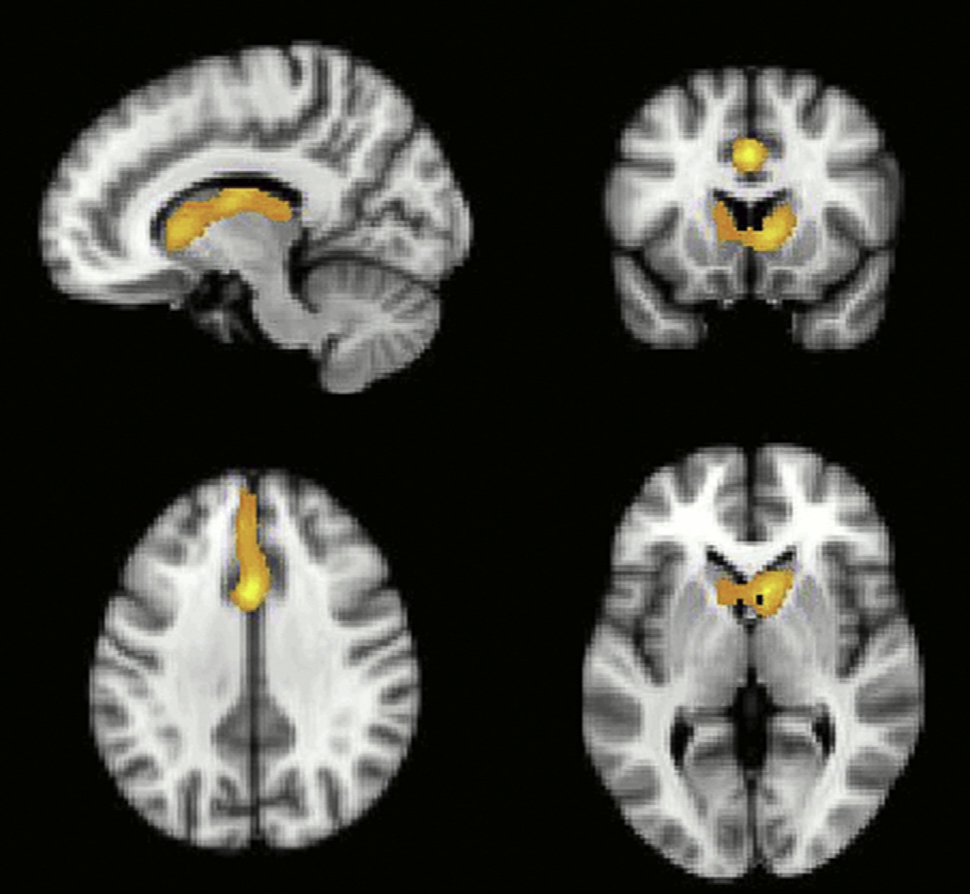
**Activation during ‘working out’ versus ‘known correct’ events contrasted across the PD and matched control groups**. The PD group showed significantly lower activation in the caudate bilaterally and the anterior cingulate cortex. (Initially thresholded voxel-wise at *p* < .05, then FWE cluster corrected at *p* < .05).

However, in contrast to the study by Hampshire and Owen (2006) in young controls, but in line with the study Hampshire et al. (2008) in older controls, there was no significant activation in the anterior insular/inferior frontal operculum (AI/FO) for this contrast. When the PD and control groups were compared directly, significant hypoactivation in patients was observed within the caudate nucleus bilaterally and the anterior cingulate (Fig. 8).

#### 3.2.2 Extradimensional switching

Switches in the focus of attention between stimulus types (ED) were then compared with switches within stimulus type (ID). In contrast to the findings of Hampshire and Owen (2006), no significant effects were observed when collapsed across groups for this contrast at the whole brain corrected threshold. Due to the strong prior prediction of AI/FO activation during ED switching in this task (Hampshire & Owen, 2006; Williams-Gray et al., 2008) we re-examined a 10 mm regions of interest (ROIs) based at the previously reported peak activation foci (analyses conducted using the MarsBaR ROI toolbox). This analysis revealed no significant ED versus ID effects in the ROI analysis (all *p* > .1 one tailed). There were also no significant between group effects for this contrast in the cluster corrected or ROI analyses.

#### 3.2.3 Reversal learning

**Fig. 9:**
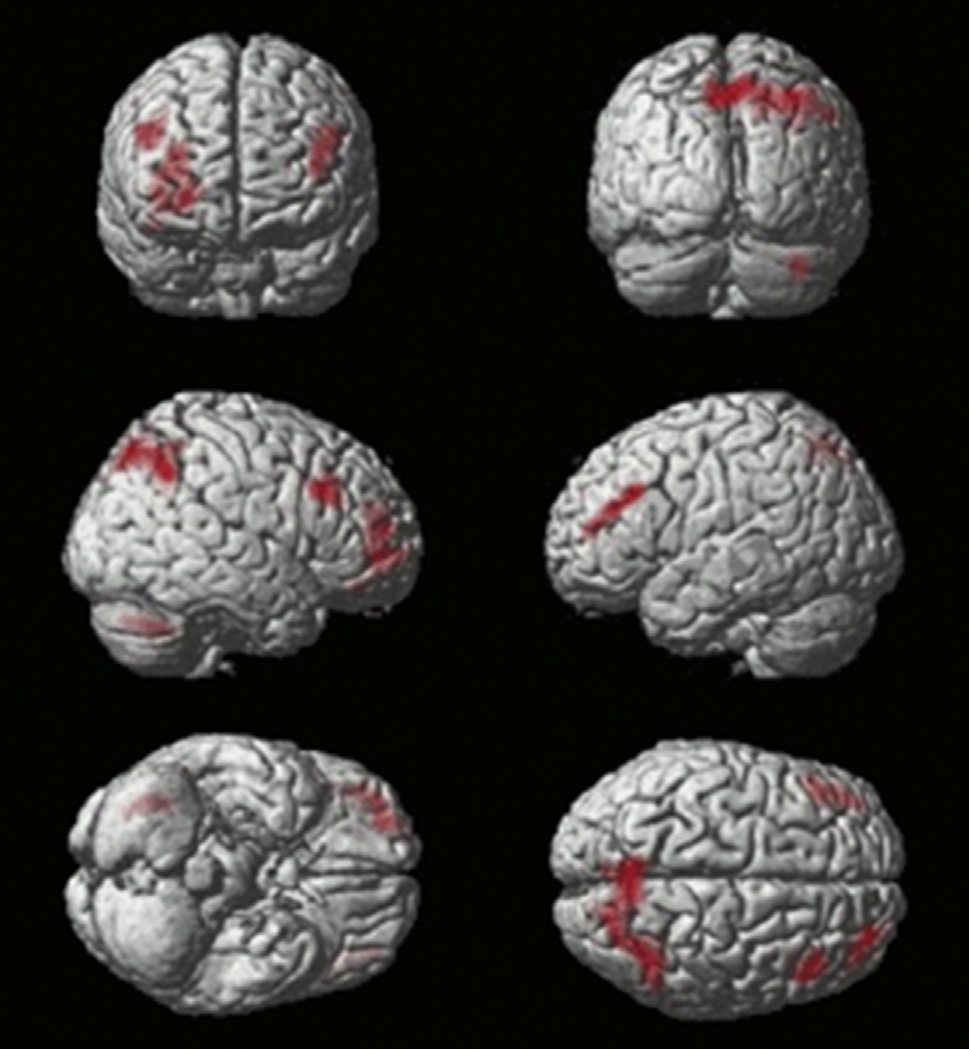
**Activation during ‘reversal’ versus ‘set-change’ events collapsed across all participants (*N*** = **34)**. (Thresholded voxel-wise at *p* < .01 with false positives controlled using cluster correction at FWE *p* < .05 for the whole brain mass in SPM5).

**Fig. 10:**
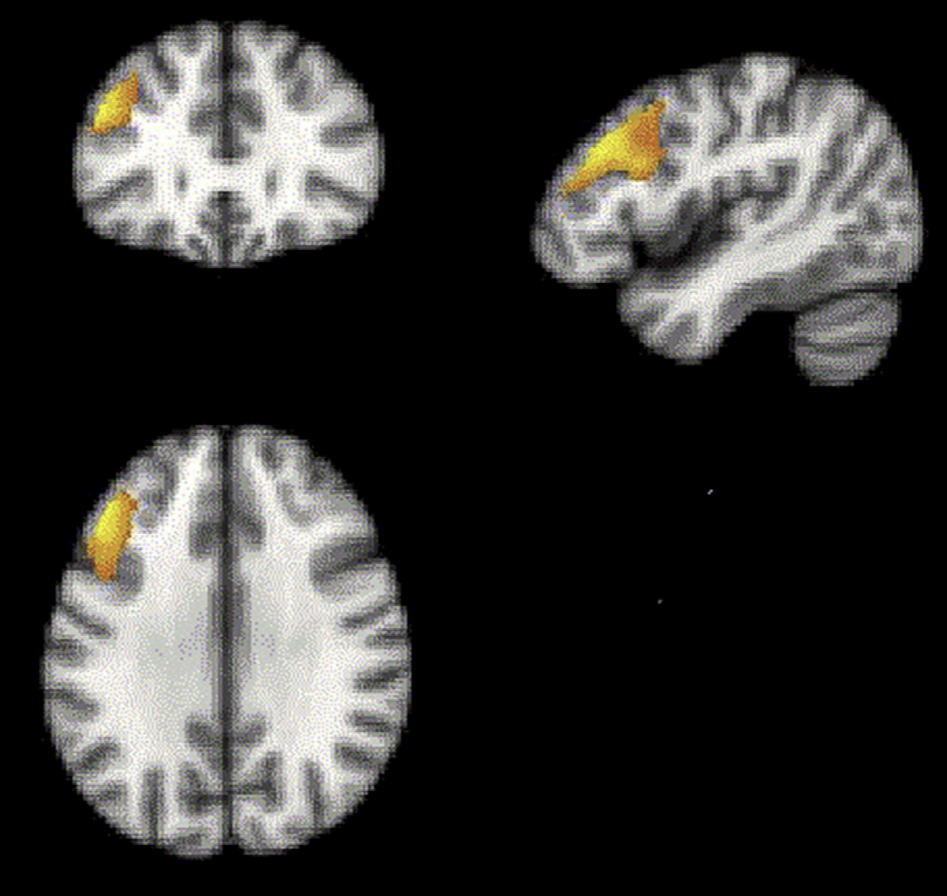
**Activation during ‘reversal’ versus ‘set-change’ events contrasted across the PD and matched control groups**. PD patients showed significantly weaker activation within the right IFS. (Initially thresholded voxel-wise at *p* < .05, then FWE cluster corrected at *p* < .05).

The first switches in selection following a change in reward contingency were contrasted with those due to stimulus set change in order to examine the reversal-learning component of attentional shifting. In line with Hampshire and Owen (2006), activation was observed in the PPC, the lateral orbitofrontal cortex (LOFC) and the IFS (Fig. 9). Cross group analyses revealed a significant cluster of hypoactivation within the right IFS in PD patients (Fig. 10).

#### 3.2.4 Feedback valence

In line with the study by Hampshire and Owen (2006), contrasting responses that lead to positive feedback minus responses that lead to negative feedback generated a cluster of activation within the medial orbitofrontal cortex (MOFC) (−2 40 −14, *t* = 4.75, *p* < .05; FWE corrected for a 15 mm sphere based) on the previously reported activation coordinates. This contrast generated no significant activation differences between the PD and the control groups.

#### 3.2.5 Effects of medication on PD group performance

As a further control, l-dopa dose was correlated with activation for the well-powered contrast of solution search versus routine responding in the PD group. There were no significant effects for this analysis anywhere in the brain.

Supplementary Fig. 11 depicts the effects of aging on brain activations examined separately in the controls and the patients with PD group for the well-powered contrast of solution search versus routine responding.

## 4 Discussion

In this study, we used an fMRI-optimised variant (Hampshire & Owen, 2006) of the classic ID/ED attentional set-shifting paradigm (Roberts et al., 1988) to further refine our understanding of the executive deficits that commonly occur in PD and normal aging. Our approach, which focused on the participant's chosen responses as opposed to the experimenter imposed conditions, allowed the individual's problem-solving strategy to be scrutinized in greater detail than has previously been possible. Based on the previous results (Williams-Gray et al., 2008) we expected to see abnormalities in PD within the networks underlying either reversal learning or extra-dimensional set-shifting. In fact, when related to the results of our previous study in young healthy controls (Hampshire & Owen, 2006), the current study revealed, that both PD patients and normally aging controls were impaired at performing the ID/ED set-shifting task. However, the finer grained behavioural characteristics and the functional anatomical bases of the executive impairments were quite distinct in PD and normal aging. More specifically, the behavioural impairment observed in PD patients was a consequence of generally slower learning of the task relative to age-matched controls (Kwak, Muller, Bohnen, Dayalu, & Seidler, 2010). This unexpected learning impairment was accompanied by hypoactivation within brain regions that are closely associated with rule learning, including the striatum bilaterally, the ACC and the right IFS. By contrast, whilst the older adults had reached their behavioural asymptotes by the start of the first task block, they were still markedly inefficient when eliminating distractors during the search for the target object. Supplementary analysis has revealed that this age-related impairment when identifying the optimal response was accompanied by hypoactivation within a set of brain regions that are closely associated with attention and short-term memory, including the AI/FO bilaterally and the pre-supplementary motor area (preSMA) (Supplementary Fig. 11).

### 4.1 The neural basis of set-shifting deficits in PD

Although the PD patients' overall performance in the second block was matched with controls, it was accompanied by hypoactivation within the IFS, striatum and ACC. Furthermore, finer resolution behavioural differences between the PD group and the control group were still evident. Specifically, while neither groups exhibited a significant advantage for IDS over EDS in terms of number of errors committed before the target was identified, only the age-matched control group demonstrated significant temporal costs of moving attention between perceptual dimension (ED) as compared to moving attention within perceptual dimension (ID). The previous study in young controls (Hampshire & Owen, 2006), implicated the AI/FO and ACC in ED switching. Consequently, the lack of an ED versus ID difference in RT is in accordance with the observed ACC hypoactivation in PD patients. Neither group displayed impaired reversal performance as indexed by error rates and reaction times. This result implies that contingency learning and behavioural inhibition were relatively preserved in PD. However, while, lateral and medial regions of the orbitofrontal cortex appeared to be functionally normal in PD and age matched control groups when the reversal contrast was examined, the patients group did show a significant cluster of hypoactivation within the right IFS (Fig. 10). The current task design used an absolute as opposed to a probabilistic contingency. Absolute contingencies make the change in stimulus-reward rule relatively unambiguous and consequently, it is important not to rule out the possibility that behavioural reversal learning deficits might be evident in the PD group when using more sensitive probabilistic designs (Hampshire, Chaudhry, Owen, & Roberts, 2012).

The observed dissociation between preserved and impaired functions of discrimination learning in the PD group corresponds well with theories that suggest that these processes are hierarchically organised. For example, Mackintosh (1983) proposed a two-stage model of animal discrimination learning, according to which the animal first identifies and selectively attends to the salient perceptual dimension and then associates its particular exemplars with reinforcement. This two stage processing account is supported by neuropsychological and functional neuroimaging evidence suggesting that neural mechanisms responsible for inhibitory control of higher-order switching between abstract task rules and lower-order switching between concrete objects are distinct (Cools et al., 2004, Dias et al., 1996, Dias et al., 1997, Roberts and Wallis, 2000). Thus, a lack of advantage for IDS over EDS observed in the PD group in the current study implies that the subjects were not attending selectively to a particular dimension, i.e., they tended not to form an attentional ‘set’ to the previously relevant dimension (Fallon, Hampshire, Barker, & Owen, 2016). This finding contrasts markedly with a number of previous neuropsychological reports that have shown that PD patients are more impaired when an ED shift is required, as compared to an ID shift (Gauntlett-Gilbert et al., 1999, Lewis et al., 2005, Owen et al., 1992, Owen et al., 1993, Roberts et al., 1988, Slabosz et al., 2006).

It is important to note however, that there is an essential distinction between the ED switching manipulations in the current investigation and that used in these classic studies. Specifically, the ED switches that were required in previous studies were typically novel, one off manipulations, whereas in the paradigm used here, many ED switches were required between two well-established stimulus categories. The current design allowed us to assess whether the strategy that an individual applies when solving a routine executive task includes organising stimuli by perceptual category. By contrast, the ED switch in classic paradigms such as the CANTAB IDED task, requires the participant to work out that such a manipulation is even possible within the context of the task and to identify which perceptual dimensions may potentially be relevant. If one posits a role for the IFS in rule learning and rule processing (Hampshire et al., 2016), then it makes sense that an IFS deficit would lead to poor ED performance on the first novel ED switch because a greater level of reasoning is required. Subsequently, dealing with trials on a more routine basis, a deficit in reasoning and rule processing could lead to a strategy that is composed of fewer sub-rules and consequently an apparent lack of attentional set (Fallon et al., 2016). Thus, the results of the current study and those that used classical ED manipulations are not discrepant when considering differences in the novelty of the ED manipulations.

Moreover, recent evidence suggest that reduced set-formation is evident in sub-groups of patients with PD according to their putative level of dopamine in the PFC (Fallon et al., 2013a, Fallon et al., 2015, Williams-Gray et al., 2008). Indeed, a pattern of behaviour similar to that observed in the PD patients in the current investigation was reported previously in studies that used the same paradigm to investigate the modulatory role of the COMT val158met polymorphism, which is known to have a marked effect on frontal-lobe dopamine levels, on attentional set formation in PD (Fallon et al., 2013b, Fallon et al., 2015, Williams-Gray et al., 2008). Specifically, these studies have revealed a difference in the ID/ED response patterns typical for val/val and met/met homozygotes, suggesting that the two groups adopt different problem solving strategies. Thus, val/val individuals make fewer errors when ID shifting than when ED shifting, adopting a strategy similar to young healthy controls (Hampshire & Owen, 2006). In contrast, met/met homozygotes exhibit no IDS advantage over EDS, a pattern that is similar to the PD patient group investigated in the current study. Although this strategy seems ‘abnormal’, it was not detrimental in terms of the overall number of errors committed before the target was identified, and in fact it seems to remedy the ED shifting impairment commonly observed in PD. In close concordance with the current results, the deficit in attentional set formation in met/met homozygotes was also associated with hypoactivation within the IFS, although it was strongest in the ‘working out’ phase of the task in those previous studies (Williams-Gray et al., 2008).

Hence, it seems plausible that the ‘abnormal’ strategy observed in PD patients when solving visual discrimination problems in the current and related studies (Fallon, Hampshire, Williams-Gray, Barker & Owen 2013; Fallon et al., 2016, Williams-Gray et al., 2008) reflects significantly compromised functions of the IFS network. Taken together, these results suggest that the pattern of attentional shifting impairments in PD may be more complex and heterogeneous than previously thought. Indeed, the results reveal that attentional set-shifting deficits in PD are likely related not only to a paucity of attentional set, but also to the adoption of an abnormal strategy while solving visual discriminations, with a concomitant hypoactivation within a set of regions including ACC, the caudate nucleus and IFS.

### 4.2 The neural basis of learning deficits in PD

Behaviourally, the most notable deficit in the PD patients was the rate at which they acquired the task. They found it particularly difficult to learn how to approach the task at the most general level, as evidenced by the fact that they still performed poorly in the first block of scanning acquisition. This slowed learning of the task was evident despite extensive pre-training prior to entering the scanner and provided a marked contrast to the age-matched control group who performed equivalently well in both blocks. Once they had acquired the task in the second scanning block the PD patients still identified fewer targets than age-matched controls, although both groups performed at the same level in terms of the mean number of responses made before the target was identified and they did not significantly differ in terms of number of specific error types (ID or ED, switch due to contingency or set-change). Our finding of comparable levels of task performance of the patients and controls is in agreement with several previous studies on set-shifting in PD (Fallon et al., 2016, Gerrits et al., 2015).

In line with Hampshire and Owen (2006), the fMRI data collapsing across groups revealed a significant solution search related activity bilaterally in the IFS and the PPC (Fig. 7). However, in contrast to the Hampshire and Owen (2006) study in young controls, there was no significant activation in AI/FO for this contrast. When the fMRI data from the PD and the age matched control were compared during the solution search phase of the task, significantly lower activity was observed in the patients in a set of regions that included the caudate nucleus and ACC (Fig. 9), commensurate with the established role for these structures in attentional set-shifting and rule-learning. Our finding is in agreement with recent studies indicating decreased activity of these regions accompanying attentional set-shifting (Nagano-Saito et al., 2014) or working memory (Ekman et al., 2012) performance in the de novo PD patients with mild cognitive impairment. Furthermore, many neuroimaging studies have implicated the caudate nucleus in set-shifting tasks like the Wisconsin Card Sorting Task and its variants (Monchi et al., 2001, Monchi et al., 2006, Stewart et al., 2001), although it has been suggested that activations of these regions may be related rather to the complexity of the set-shifting paradigms than to set-shifting activity itself (Witt & Stevens, 2013). In a review, Grahn, Parkinson, and Owen (2008) have proposed that the caudate nucleus contributes to goal-directed learning (i.e., behaviour that is guided by response-dependent feedback), as it is sensitive to action contingencies and the evaluation of subsequent outcomes. Collins, Wilkinson, Everitt, Robbins, and Roberts (2000) investigated the effects of DA lesions restricted to the caudate nucleus on cognitive function in primates. The results demonstrated that reductions in DA activity within the caudate nucleus impaired the ability to learn a visual discrimination that required the re-engagement of a previously relevant attentional set. Overall, the profile of set-shifting impairments seen in the patients with PD in the current study and in other studies using ID/ED paradigms (e.g., Downes et al., 1989, Owen et al., 1991, Owen et al., 1992, Owen et al., 1993) is clearly much more general than that described by Collins et al. (2000). However, it is likely that these behavioural differences are a consequence of the widespread loss of DA throughout the striatum, and not just within the caudate nucleus, observed even at the early stages of PD. Our study revealed strong hypoactivation within both the dorsal striatum and the ventral striatum in the group of the patients, and this result corresponds well therefore, with the observation of a profound learning impairment (Hampshire et al., 2016, MacDonald and Monchi, 2011, MacDonald et al., 2011).

Summarising, the surprising result of the current study was an observed ‘meta-level’ effect, whereby patients were impaired at learning the rules of the task overall. This result accords particularly closely with the observation that PD affects model-based reinforcement learning (Fallon et al., 2016, Sharp et al., 2016, de Wit et al., 2011, de Wit et al., 2012). These types of fronto-striatal mechanisms that have been examined by O'Doherty, 2004, O'Doherty et al., 2003, O'Doherty et al., 2007 and more recently ourselves in healthy controls (Hampshire et al., 2016). Recent work suggests that model-based learning may involve dopamine modulation, contributing to the learning impairment observed in PD (Sharp et al., 2016).

### 4.3 The neural basis of routine-problem solving deficits in normal aging and in PD

In a supplemental correlational analysis, the effects of aging on brain activations were examined separately in the control and PD groups for the well-powered contrast of solution search versus routine responding (Supplementary Fig. 11). In contrast to the cross group difference observed in the previous study (Hampshire et al., 2008), in the current study a broader set of brain regions was shown to be affected by age, i.e., not just the AI/FO, but also the IFS and PC. This set of areas is broadly associated with multiple demand regions (Duncan, 2010, Hampshire et al., 2012b). This finding fits well with the strategy deficiency hypothesis outlined above (Fallon et al., 2016).

Interestingly, the effects of aging observed in a supplementary analysis conducted in the PD group alone were qualitatively different in both behavioural and neural terms. Notably, in the PD group, age was only marginally correlated with the Hoehn and Yahr scale (*r* = .51, *p* = .065) and unrelated to any other variable reflecting clinical status of the patients. This result is likely to reflect the fact that the patients were selected according to stringent criteria in order to ensure clinical homogeneity, thereby precluding age-related differences in the clinical status of the patients. In the PD group, there was a significant negative correlations between age and activation within the caudate nucleus, thalamus, precuneus and mid DLPFC. This accords well with the idea of progressive frontostriatal impairments in PD that are distinct from those observed in normal aging (Hughes, Barker, Owen, & Rowe, 2010), although further research is required.

One of the limitations of the current study was the fact that all participants with PD remained on their prescribed l-dopa medication regimes, constraining the possibilities of testing any predictions regarding medication directly. In contrast to Williams-Gray et al. (2008), the current study revealed only the subtle behavioural effects and no significant neuronal effect of l-dopa. Further work is required to unravel medication effects on attentional-set shifting performance and concomitant neural activity in PD.

## Funding

This work was supported by Wellcome Trust (grant number: WT072378MA) to AG and AMO and a Canada Excellence Research Chair award to AMO.
