# Supplementary material for: Normal aging and Parkinson's disease are associated with the functional decline of distinct frontal-striatal circuits
Source: Cortex. 2017 Aug;93:178–92. doi: 10.1016/j.cortex.2017.05.020 (PMC5542042; doi:10.1016/j.cortex.2017.05.020)

***Supplementary materials***

**Supplementary Figure 3a. Effects of PD on error rates.**This figure illustrates the effects of (A) dimension change (ID, ED) and (B) reversal of reward contingency (set change, reversal) on mean number error rates compared across the patients with PD and the matched control group (CS) for block one and block two of the experimental task. Bars represent standard error of the mean (p=0.133).


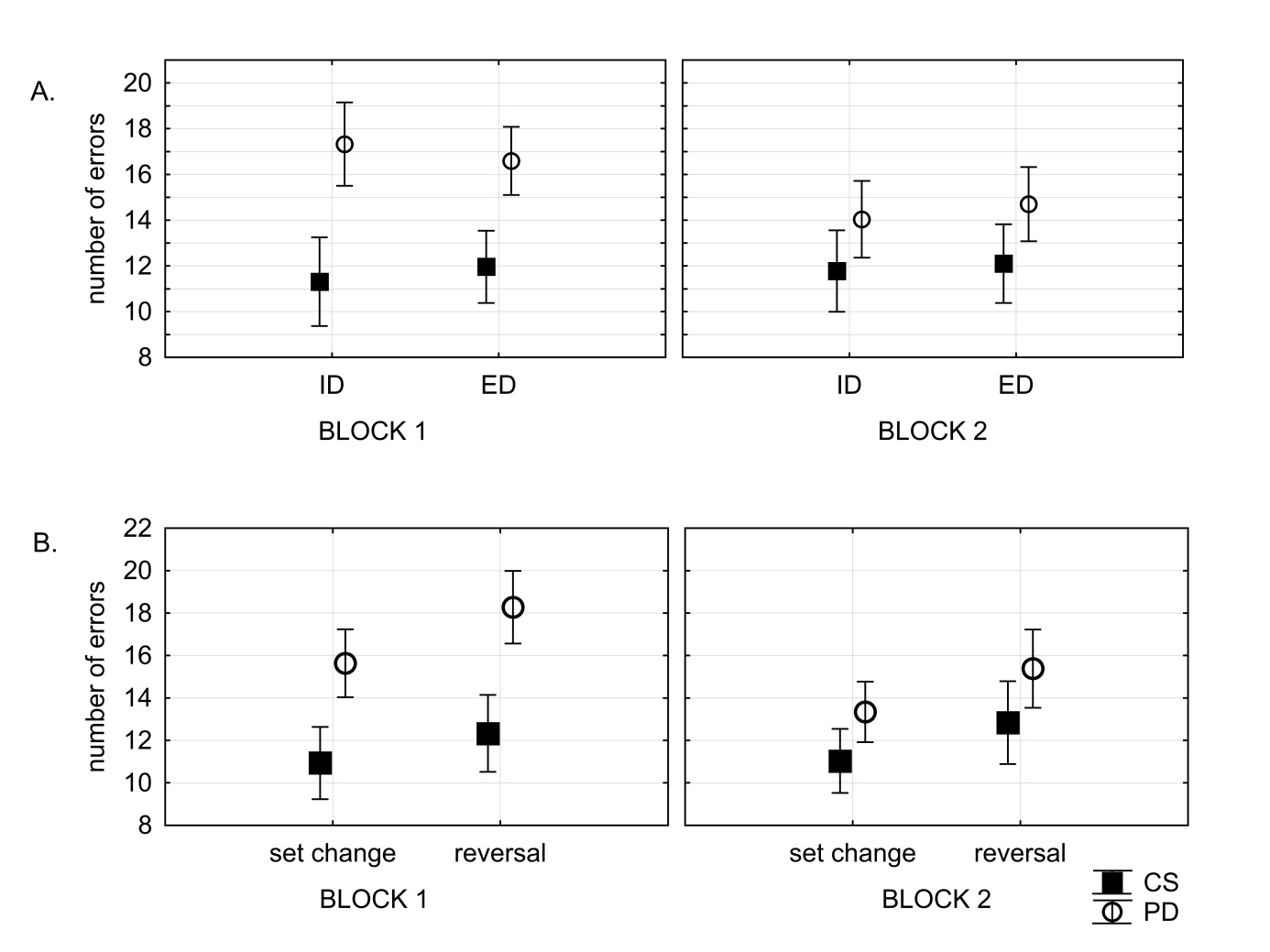


**Supplementary Figure 5a**. **Effects of PD on response times.** This figure illustrates the comparison of groups (patients with PD versus age matched controls) on reaction times for switch types for the second block of the experimental task (p=0.726).


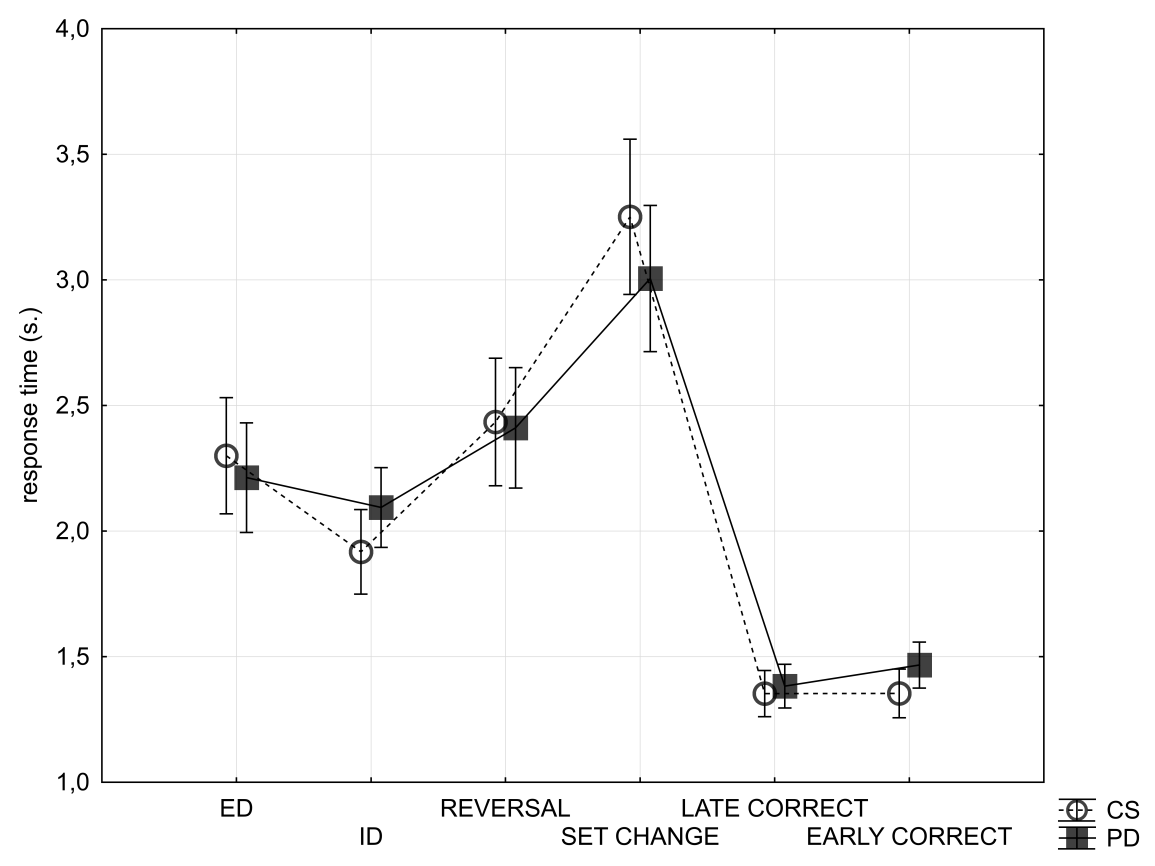


**Supplementary Figure 6A. Effects of L-dopa on response time**. This figure illustrates the effects of L-dopa on different type of response events for the patients with PD for both the first and the second block of the task (p=0.971). L-dopa dose was broken down by median-split for visualization purpose only. Bars represent standard error of the mean.


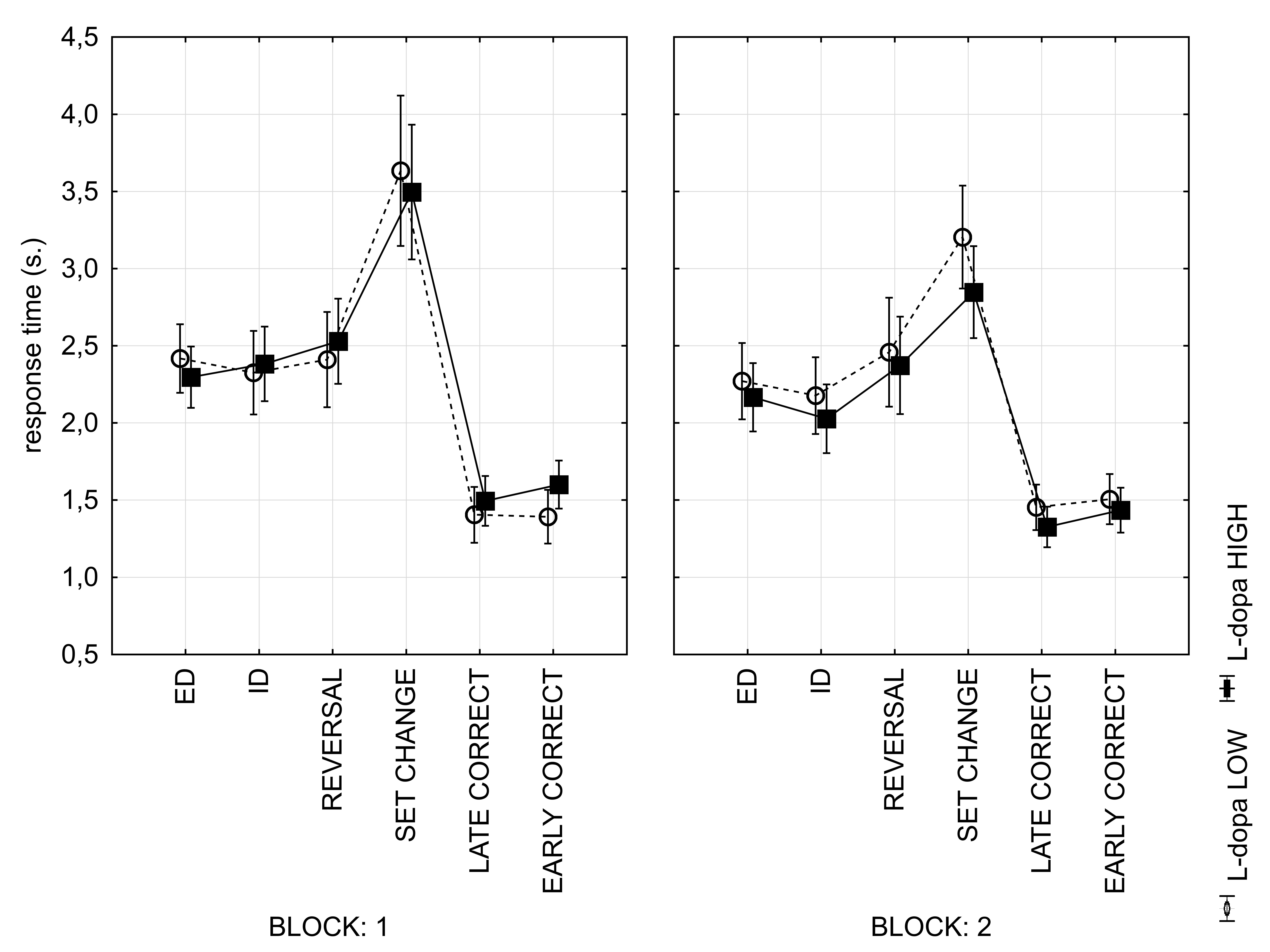


**Supplementary Figure 11.** ***Effects of aging on brain activations observed in normal aging and PD pathology***. In a supplemental correlational analysis, the effects of aging on brain activations were examined separately in the control and PD groups for the well-powered contrast of solution search vs. routine responding. In the control group, there was a significant negative correlation between age and activation within a set of areas broadly associated with multiple demand (MD) regions (Duncan, 2010; Hampshire, Highfield, Parkin, & Owen, 2012), including AI/FO, IFS and PC (shown in blue). In the PD group, there was a significant negative correlations between age and activation within the caudate nucleus, thalamus, precuneus and mid DLPFC (shown in yellow). (Initially thresholded voxel-wise at p<0.05, then FWE cluster corrected at p<0.05).


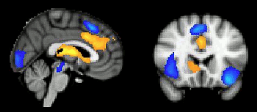

Supplement: Supplementary file 1 [file mmc1.docx]
